# Design and Demonstration of a Hybrid FES-BCI-Based Robotic Neurorehabilitation System for Lower Limbs

**DOI:** 10.3390/s25154571

**Published:** 2025-07-24

**Authors:** Kasper S. Leerskov, Erika G. Spaich, Mads R. Jochumsen, Lotte N. S. Andreasen Struijk

**Affiliations:** 1 The Neurorehabilitation Robotics and Engineering Group, 9260 Gistrup, Denmark; 2 The Center for Rehabilitation Robotics, 9260 Gistrup, Denmark; 3 Department of Health Science and Technology, Aalborg University, 9260 Gistrup, Denmark; 4 Neurorehabilitation Systems Group, 9260 Gistrup, Denmark; 5 Neural Engineering and Neurophysiology, 9260 Gistrup, Denmark

**Keywords:** brain–computer interface, stroke, functional electrical stimulation, robotics, neurorehabilitation, movement-related cortical potential, spinal cord injury, biomedical engineering

## Abstract

Background: There are only a few available options for early rehabilitation of severely impaired individuals who must remain bedbound, as most exercise paradigms focus on out-of-bed exercises. To enable these individuals to exercise, we developed a novel hybrid rehabilitation system combining a brain–computer interface (BCI), functional electrical stimulation (FES), and a robotic device. Methods: The BCI assessed the presence of a movement-related cortical potential (MRCP) and triggered the administration of FES to produce movement of the lower limb. The exercise trajectory was supported by the robotic device. To demonstrate the system, an experiment was conducted in an out-of-lab setting by ten able-bodied participants. During exercise, the performance of the BCI was assessed, and the participants evaluated the system using the NASA Task Load Index, Intrinsic Motivation Inventory, and by answering a few subjective questions. Results: The BCI reached a true positive rate of 62.6 ± 9.2% and, on average, predicted the movement initiation 595 ± 129 ms prior to the MRCP peak negativity. All questionnaires showed favorable outcomes for the use of the system. Conclusions: The developed system was usable by all participants, but its clinical feasibility is uncertain due to the total time required for setting up the system.

## 1. Introduction

In the rehabilitation of neurologically injured individuals suffering from, e.g., a spinal cord injury (SCI) or a stroke, early rehabilitation has been shown to be beneficial [1,2,3,4]. However, for severely affected individuals, early physical therapy may be delayed as their general condition requires them to remain bedridden [5]. This delay can have detrimental effects on the patients’ ability to perform activities of daily living [6], as the patients’ capacity for neural improvement is particularly primed in the early days following their injury [2,7]. Thus, delaying training in the early days following injury may constitute a lost opportunity to gain functional improvement [6].

For bedridden patients, passive exercise may be administered to prevent circulatory problems and the deterioration of tissue properties and range of motion [5,8]. By imposing a functional movement to the impaired limb while bedridden through, e.g., robotics or functional electrical stimulation (FES), the motor function of the patient may additionally be improved [3]. However, for these interventions to be optimal, it is important that they are paired with active participation of the injured individual, as this is important to facilitate motor learning [9,10,11].

Robotics have become increasingly available in neurorehabilitation and may provide patients with a longer duration of therapy with more repetitions while putting less strain on healthcare professionals [3,12,13]. Most of the commercially available robotic devices for lower limb exercising focus on out-of-bed exercises, e.g., Lokomat [14], HAL [15], and EksoGT [16], although robotics for bedridden individuals also have been developed, e.g., the Toe-Up! for ankle-dorsiflexion [5] and the NEUROBike for ellipsoidal exercises inspired by activities of daily living [17]. However, while using the systems for in-bed exercising, patients remain passive and their participation is not actively needed [5,17], which is essential for an improved rehabilitative outcome [9,10,11].

FES is an alternative to robotics to support exercising for bedridden individuals, where muscle activation is facilitated with electrical stimulation, which has been shown to improve long-term motor and sensory recovery [18,19]. However, similar to robotics, FES systems alone do not actively consider the patients’ participation in the exercise, although their muscles are active due to the electrical stimulation. Additionally, FES inherently induces rapid fatigue [20,21], and it is hard to control in order to produce precise repetitive movements [22].

By combining robotic and FES systems, it is possible to mitigate the downsides of the respective technologies. In a combined system, FES can ensure the active engagement of muscles, and robotics can lessen the fatigue induced by FES and provide better force control of the produced movement [23,24]. It has additionally been suggested that combining robotics and FES may be an effective way to increase the effectiveness of robotic interventions [25]. These hybrid systems have previously been constructed [26], e.g., by combining a Lokomat with FES [27] or cycling with FES [28]. However, these hybrid systems focus mainly on out-of-bed exercises, which are inapplicable to severely affected individuals who remain bedridden.

Petersen et al., 2020 introduced a hybrid robotic rehabilitation system, which paired the administration of robotic mobilization, FES, and the active participation of users [29]. This was achieved by using electromyography (EMG) to determine when to administer FES based on a single-threshold algorithm [29]. However, for severely injured individuals with little to no voluntary remaining leg muscle activity, this method would most probably not work, due to insufficient EMG signals.

Alternatively, active participation of severely affected individuals may be accomplished by utilizing a brain–computer interface (BCI). BCIs are systems that translate the brain activity of a user into commands useable by programmable devices such as robotics and FES systems [30]. BCIs are, in most cases, capable of utilizing brain signals of people with a neurological injury [31,32,33]. When these signals are used by a BCI to detect movement initiation and couple it with robotics-facilitated movement actuation, the effect of the robotic therapy is promoted [30,32]. Similarly, using a BCI-triggered FES system has been found to increase the effect of the exercise [33,34]. To the best of the authors’ knowledge, no hybrid robotic–FES system for bedridden patients incorporates the use of a BCI for triggering FES.

Robotics, FES, and BCI have previously been combined as FES–robotics [26,27,28], BCI–robotics [30,32], and BCI–FES [33,34], with applications in rehabilitation. This study aims to combine all three technologies in a novel hybrid robotic–FES rehabilitation system utilizing BCI-triggered FES and to demonstrate the technical performance and feasibility of the system. The performance of the system is assessed as the BCI true positive rate and latency of detections, while the technical feasibility of the system is assessed as the amount of completed exercises, the required time to use the system, and the participants’ perception of the system. The novelty of the system is the combination of an end-effector robot and the BCI-controlled administration of FES, for facilitating lower limb exercises while in a supine position. The system is developed for severely impaired patients with little to no voluntary muscle activity and facilitates early, repetitive, and resistive functional exercise with active participation in a bedridden setting, without any strain on healthcare professionals during exercise. Here, we present the system design and the first test of the hybrid robotic–FES–BCI rehabilitation system in healthy participants.

## 2. Materials and Methods

**Figure 1:**
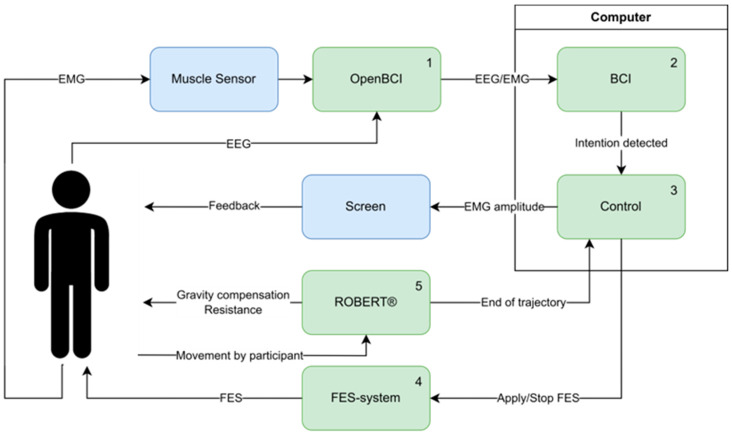
An overview of the developed system and additional experimental hardware. System components are highlighted in green and additional experimental hardware in blue. The numbering indicates the progression of control signals/activation in the system during use. (1) EEG and EMG were recorded from the participant through the OpenBCI Cyton board (with a Myoware Muscle Sensor for EMG) and transmitted wirelessly through Bluetooth to a computer. (2) Once on the computer, the EEG and EMG were streamed through a Lab Streaming Layer from the OpenBCI firmware to MATLAB, where the BCI and additional system control were implemented. The EEG was recorded as input for the BCI, and the EMG provided feedback through a screen where the EMG amplitude was shown. The BCI was trained to identify the time of peak negativity in the MRCP, indicating that the participant had the intention to move. (3) Upon detecting the intention to move, the BCI triggered the administration of FES (4) to aid the participant in completing the exercise. (5) During the FES-induced movement of the participant’s leg, ROBERT^®^ provided resistance to the movement/exercise and gravity compensation. When the exercise trajectory was completed, ROBERT^®^ sent a notification to the computer. This signal was used to turn off the FES.

The novel system presented in this study was designed to facilitate the active participation of users unable to produce voluntary muscle contraction, by utilizing a BCI for interpreting the users’ intention of performing exercise and subsequently aiding them in completing the exercise using FES. See an overview of the system and additional experimental hardware in Figure 1.

### 2.1. System Design

The robotic–FES–BCI system was composed of ROBERT^®^ (by Life Science Robotics ApS, Aalborg, Denmark), an FES system (NoxiSTIM, by JNI Biomedical, Aalborg, Denmark), and a BCI system (Cyton Biosensing Board, by OpenBCI, Brooklyn, NY, USA) + EEG-cap (NuAmp, by Compumedics, Freiberg, Germany) and a PC running the control of the FES system and the BCI.

#### 2.1.1. Hardware

**Figure 2:**
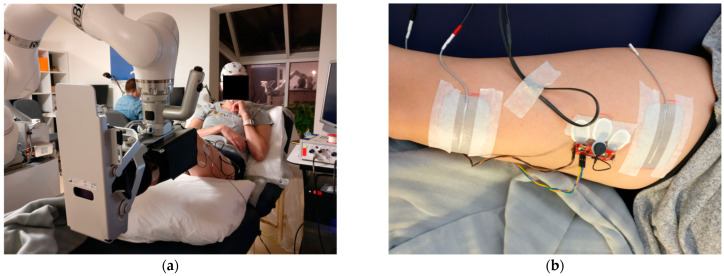
(**a**) A participant’s leg supported by ROBERT^®^ in the custom foot-brace. (**b**) Positioning of stimulation electrodes and EMG electrodes on the rectus femoris.

ROBERT^®^ is an end-effector robot based on the Kuka LBR Med 14 R820, with a pose precision of ±0.15 mm and maximum payload of 14 kg [35]. ROBERT^®^ is used for lower-limb rehabilitation (see Figure 2a) and helps the user to complete a programmed exercise trajectory [29], by inferring a tunnel of possible movement centered on the desired trajectory, leaving room for variation in the movement execution [36], which is beneficial for neurorehabilitation [37]. In the present study, the robot provided resistance to the exercise to be performed while compensating for gravity. Additionally, the ROBERT^®^ device was equipped with a “safe mode”, which limited the tolerated interaction forces between the limb of the participant and the robotic movement. If joint torques exceeded a set threshold, the “safe mode” would engage and lock the participant’s leg in its current position, from where the experimenter could reposition the leg in a relaxed position [36].

The FES system employed in this study utilizes two electrodes (5 × 9 cm Durastick Premium by CefarCompex, Malmo, Sweden, see Figure 2b). The stimulation consisted of rectangular monophasic pulses with a pulse duration of 250 μs and a frequency of 50 Hz. The stimulation intensity was assessed for every participant individually. The procedure for assessing the stimulation intensity is described in the Section 2.2.2. During online use, the experimenter would observe each movement performed by the participants, and in case any movements were deemed inappropriate, or the participant indicated something was wrong, the stimulation would be terminated manually, and the cause for stopping was investigated prior to continuing.

The BCI used five passive ring Ag-AgCl electrodes. Additionally, an EMG channel was added to the Cyton Biosensing Board, using a Muscle Sensor (AT-04-001 by Myoware, Advancer Technologies, Raleigh, NC, USA) for monitoring EMG during the experiment. The EEG and EMG were sampled at 250 Hz. The data sampled by the Cyton Biosensing Board were transmitted through Bluetooth to the PC.

After the EEG was transmitted to the computer, it was fed through a Lab Streaming Layer implemented in the OpenBCI GUI firmware. Then, the data were processed in MATLAB (version R2019a), where the BCI and remaining system control were implemented.

#### 2.1.2. Brain–Computer Interface

**Figure 3:**
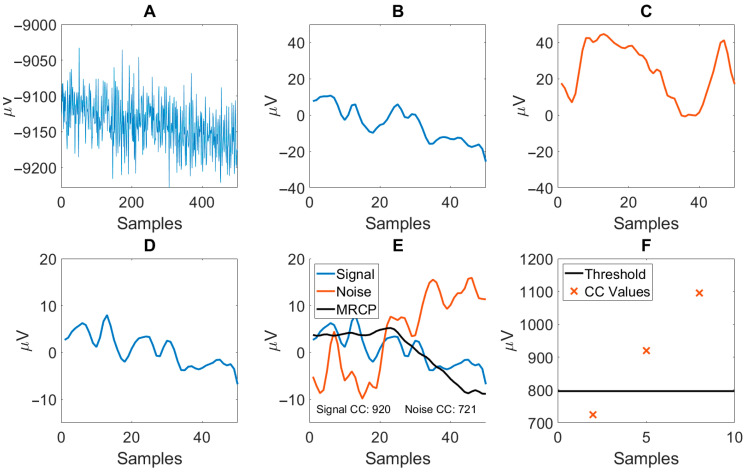
Visual representation of the processing steps required by the BCI to determine the presence of an MRCP in raw EEG data. The same EEG snippet is shown in this figure after various processing steps, unless otherwise specified. (**A**) Raw EEG. (**B**) ‘Signal’ EEG following lowpass (12 Hz) filtering, downsampling, and highpass + lowpass filtering (0.1–5 Hz). (**C**) Example of ‘noise’ EEG (not based on the illustrated raw EEG), used for calculating the coefficient of the OSF and to calibrate the threshold for the classifier. (**D**) ‘Signal’ EEG following spatial filtering. (**E**) Illustration of the ‘signal’ and ‘noise’ epochs and their respective cross-correlation (CC) values calculated using the MRCP template (‘MRCP’). (**F**) Upon detecting two out of three consecutive CC values above the identified threshold during online use, the presence of an MRCP is accepted, and subsequently FES is administered.

The BCI design of the current study was inspired by a previous study, showing acute neuroplastic effects following a single intervention session consisting of single stimulation pulses delivered to the common peroneal nerve 50 ms prior to the anticipated peak negativity of the MRCP [38] (details about the BCI are found in [39]). The various processing steps required by the BCI to translate raw EEG into a detection of an MRCP are outlined in the following and are visually presented in Figure 3.

**Figure 4:**
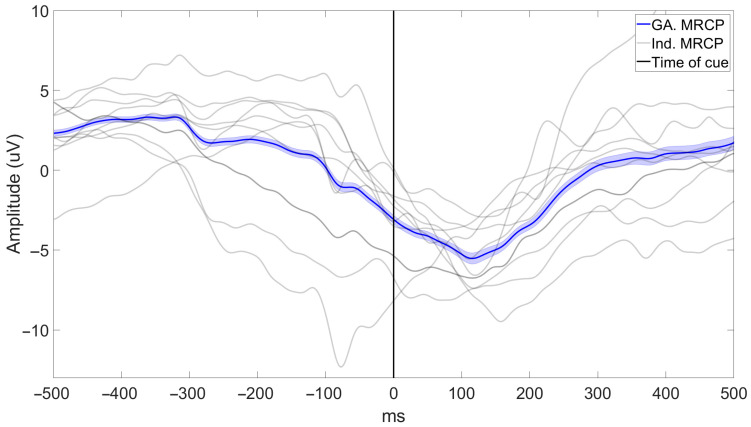
Example of the MRCPs obtained in this study. The grand average (GA.) MRCP across all participants (illustrated with standard error) and the average MRCP of each individual (Ind.) participant. Additionally, MRCPs are illustrated relative to the time of the “GO” cue (time: 0 ms, see Section 2.2.3 and Section 2.2.4) prompting the participants to perform a movement (see Section 2.2.3 and Section 2.2.4).

Preprocessing: Raw EEG data (see Figure 3A) were filtered using second-order Butterworth filters. Firstly, a lowpass filter (fc = 12 Hz) was used prior to the data being downsampled to 25 Hz. Following downsampling, the EEG data were bandpass filtered using a lowpass (fc = 5 Hz) and a highpass filter (fc = 0.1 Hz). See the filtered EEG in Figure 3B. These cutoff frequencies were chosen based on typical filter designs optimized for finding the Movement-Related Cortical Potential (MRCP, see Figure 4) to be used for identifying movement intention [40].

Spatial filtering: After filtering, 50 two-second epochs of ‘signal’ and matching 50 two-second epochs of ‘noise’ were extracted (see Figure 3C). ‘Signal’ epochs were extracted at times of MRCP (see Section 2.2.3) and ‘noise’ epochs were extracted at times two seconds prior to the MRCP. Each epoch contained data for all five EEG channels. Following extraction, epochs were removed if they violated the following:

(1) $$e_{ik}\leq \;\mu_{G}\pm 3\sigma_{G},$$

where $e_{ik}$ is the i’th sample of the k’th channel in the respective epoch, $\mu_{G}$ is the grand average amplitude of either ‘signal’ or ‘noise’ epochs, and $\sigma_{G}$ is the standard deviation of the grand average ‘signal’ or ‘noise’ epoch. Epochs were only tested according to (1) for their respective epoch type. If an epoch violated (1), it and its matching epoch (either ‘signal’ or ‘noise’) were removed to maintain a balance between the numbers of ‘signal’ and ‘noise’ epochs.

Afterwards, the remaining epochs were processed to obtain the coefficients of a spatial filter referred to as the “Optimized Spatial Filter” (OSF) [39]. Briefly described, the filter is constructed initially as a small Laplacian filter with five filter coefficients, which are tuned by using epochs of ‘signal’ and ‘noise’ to maximize the signal to noise ratio between the two sets of epochs [39]. A more in-depth explanation can be found in the paper by Niazi et al., 2011 [39]. The resulting filter coefficients of this procedure were used to process the data further for BCI calibration and were applied to the data during online testing.

Feature extraction: The spatial filtering employed combined the five EEG channels into a single channel of EEG to be processed (see Figure 3D). From this, the mean ‘signal’ epoch was calculated, using the previously identified ‘signal’ epochs, and denoted a “template of MRCP.” The template included samples from the MRCP peak negativity (PN) and two seconds prior (see Section 2.2.3). Subsequently, a single feature from the remaining ‘signal’ and ‘noise’ epochs was calculated as the max cross-correlation between the epoch and the MRCP template (template matching, see Figure 3E). This was the only feature of the BCI.

Classifier: Finally, a threshold to separate ‘signal’ and ‘noise’ epochs, based on the MRCP template cross-correlation values, was determined. This was achieved by comparing the accuracies determined by brute-forcing through preset threshold values. The threshold values used in the present study were −ex to ex, with x values from 0 to 100, in steps of 0.1. These threshold values were determined to be sufficient to produce a reliable ROC curve, even with outlier performance, in pilot studies. The final selected threshold was the highest threshold with a false positive rate lower than 0.2. After the threshold had been found, the BCI was fully calibrated for online use. In pilot studies, it was found that the described method produced the most reliable BCI performance; however, the BCI was even more robust if the identified threshold for detection was adjusted by a factor of 1.5. Hence, the identified threshold value was corrected by a factor of 1.5 prior to online use.

Online classification: During online classification, a cue was used to prompt the participant to initiate an exercise. The BCI was only active from a second before to a second after the expected action, thus preventing inappropriate administrations of FES when the participant was unprepared. Classifications were made every 120 ms in this window, on the current and the two previous segments of EEG. If two of these segments were classified as containing an MRCP (see Figure 3F), a detection was accepted, and a trigger was sent to activate FES. If at any point the online performance decreased due to mental fatigue, concentration issues, changes in ambient noise, etc., the investigator could adjust the factor multiplied by the threshold. This change was made in increments or decrements of 0.1 and depended on the investigators’ experience as to when a change was appropriate. Generally, after three unsuccessful attempts at triggering the BCI, the threshold factor was decreased. For each unsuccessful attempt following the adjustment, additional adjustment was made until a successful trigger was generated. Additionally, if participants expressed that “they did nothing” and still received FES, or FES administration was found to be consistently ‘early,’ the threshold was increased.

### 2.2. Experimental Procedure

The evaluation of the system was conducted over a single session in an office-like workplace (non-lab setting). To minimize noise contamination of signals in the system, all equipment, including the robotic device, was grounded to the same source.

#### 2.2.1. Participants

Ten healthy participants (6 males; mean age 27.1 ± 4.4 years) were recruited to exercise with the system. Ethical approval was obtained from the local ethics committee (N-20190034), and an informed consent form was signed by all participants. No participant had any known neurological disease. All experimental procedures followed the Declaration of Helsinki.

#### 2.2.2. Participant Preparation

Firstly, the participant sat reclined on a bed to ensure roughly 30 degrees of hip flexion. The participant was then fitted with an EEG cap including EEG electrodes and EMG and stimulation electrodes. For an overview of the system and experimental setup, see Figure 1. The EEG electrodes were placed at positions Fz, C3, Cz, C4, and Pz, and two EMG electrodes (Ambu^®^ Neuroline 720, by Ambu, Ballerup, Denmark) serving as ground and reference for the EEG acquisition were placed on the right and left mastoid processes. Two EMG electrodes were placed on the rectus femoris in accordance with the SENIAM guidelines [41] to record EMG activity during knee extension. The two stimulation electrodes (Dura Stick Premium, 8 × 13 cm) were placed to target the proximal motor points of the rectus femoris according to Botter et al., 2011 [42].

Subsequently, the robot was attached to the participant’s leg, using a custom foot-brace that was adjusted to fit individual participants using wide adjustable straps (see Figure 2a). The investigator then programmed ROBERT^®^ to follow a trajectory comparable to a knee extension exercise by moving the participant’s leg through the desired trajectory.

Then, the resistance of ROBERT^®^ and the amplitude of the FES were calibrated following the procedure described by Leerskov et al., 2022 [43]. In summary, the resistance was set to a level that would ensure gravity compensation. Subsequently, the amplitude of FES gradually increased in increments of 2 mA to a level generating a muscle contraction capable of completing the exercise repetition at the set resistance of ROBERT^®^. If the resistance could not be overcome using an FES amplitude acceptable by the participant, the resistance of ROBERT^®^ was lowered, and the amplitude of FES was reassessed [43]. This procedure continued until satisfactory levels of resistance and FES amplitude were reached.

#### 2.2.3. BCI Calibration and Data Acquisition

After setting up the system, the BCI was calibrated. During calibration, the participants were asked to do a minor initiation of a knee extension exercise 50 times, with their leg in the starting position of the knee extension exercise, i.e., the hip and knee joint flexed to approx. 90 degrees. To standardize the participant’s effort, EMG-based feedback was used. The participant had to reach an exertion level equivalent to twice the EMG amplitude achieved during rest; this target was shown to the participant on a screen and was calculated as the mean EMG in a 10 s interval recorded at rest with the leg in the starting position of the knee extension exercise. The participant was instructed to attempt to reach this exertion level briefly, using as little movement as possible, and to relax quickly upon completing the task. For every repetition, a countdown of four cues—“3”, “2”, “1”, and “GO”—was shown to the participant on the screen, with a second between every cue. The participant was instructed to perform the movement at the “GO” cue. At this time, a trigger was sent to the computer, which would be used to calibrate the BCI (as ‘time of MRCP’). Each repetition of this task was separated by 10 s, i.e., 10 s separated two occurrences of “GO”.

#### 2.2.4. BCI-Triggered Exercise

After calibrating the BCI, exercise with the system commenced. The exercise consisted of performing as many repetitions of the knee extension exercise as possible during a 60 min session. For each repetition, a countdown was shown to the participant, similar to the one used during calibration, and the participant was instructed to do the same minor initiation at the time of “GO.” BCI detection was enabled only one second prior to “GO” and one second after. If a detection was made by the BCI, FES was activated, resulting in the participant’s leg exerting force on ROBERT^®^ and completing the knee extension exercise. While FES was being delivered, the BCI was disabled. If a detection was not made, a new countdown was shown 10 s later. If a detection was made, a new cue would be present 3 s after ROBERT^®^ returned the leg of the participant back to the starting position. Breaks were held after every 20 activations, or when necessary for the participant. After a break, the participant would indicate to the experimenter when they were ready to continue.

### 2.3. Data Acquisition

EEG, EMG, ROBERT^®^, and BCI commands were continuously logged. Likewise, the time spent setting up and using the system was noted during the experimental session to assess the time taken to set up, calibrate, and use the system. The allotted time to exercise was 60 min, but some sessions had to be shortened due to fatigue of the participant or other practicalities such as participants having to leave for personal reasons.

Finally, questionnaires on the experience of using the BCI-controlled ROBERT^®^-FES system were filled out online after the session (SurveyXact, by Ramboll, Aarhus, Denmark). The questionnaires were the NASA Task Load Index (TLX), the Intrinsic Motivation Inventory (IMI), and a subjective questionnaire (SQ).

The TLX is a questionnaire designed to assess workload based on six subscales: Mental Demands, Physical Demands, Temporal Demands, Own Performance, Effort, and Frustration [44]. For each subscale, the participant is asked to what degree, on a scale from 0 to 20 (0 = low, 20 = high), workload for the given subscale was present during the exercise. Subsequently, the participant is asked to rank which of two subscales was more demanding in completing the activity, for all possible subscale pairs. This ranking is used to calculate the adjusted subscale scores as a product between the raw score and the number of times the respective subscale was chosen as the more demanding of a pair of subscales (0 = low, 100 = high). Following this, a total task load can be calculated as the sum of adjusted subscale scores divided by 15 (0 = low, 20 = high) [44]. The IMI is a questionnaire designed to assess the intrinsic motivation of a participant in relation to an activity [45]. In this study, 29 of the total 45 questions in the IMI were used, as was previously done by Wang et al., 2018 [46]. These questions relate to four of the seven subscales of the IMI: Interest, Perceived Competence, Effort, and Tension. The participants are asked to rank to what degree they agree with each question/statement on a scale from 1 to 7 (1 = not at all true, 7 = very true). The SQ is adopted from Mazzoleni et al., 2014, who used seven subjective statements to assess the users’ perception of a newly developed therapeutic device [47]. In this study, four of the original seven statements [47] are used, as the other three are related to subjective matters only relevant to patients. The statements were as follows:

1. You felt comfortable with the exercise.
2. You did not experience pain during the exercise.
3. You got tired during the exercise.
4. You enjoyed the exercise.

The participants should note to what degree they agreed with each of the four statements on a scale from 0 to 7 (0 = not at all agree, 7 = strongly agree).

### 2.4. Data Analysis

The main outcome of this study was the performance matrixes of the BCI quantified as BCI classification TPR and the latency of BCI detections relative to the intended activation.

Calculation of the BCI TPR was achieved based on the total number of activations and the number of attempted activations. Activations were counted when FES was delivered, and attempted activations were counted as the number of cues provided to the participants to attempt activation.

The latency of the BCI’s detection relative to the intended activation was calculated as the difference between the time when the BCI detected an attempted movement and the time of peak negativity of the average MRCP. The peak negativity of the MRCP was identified as the most negative peak of the average MRCP across all exercise repetitions (where a BCI detection was made) within a bout of exercise, i.e., a new MRCP and peak negativity were found for every active session of the participants, separated by breaks. A total of three single trials across all participants had to be excluded from calculating the average MRCPs due to heavy noise contamination.

The secondary outcomes of this study were feasibility measures related to the amount of exercise repetitions completed in 60 min, the threshold factors used during the session, the time spent using the system, and the overall participant perception of exercising with the system as assessed with the TLX, IMI, and SQ.

The time spent using the system was calculated based on the timestamps noted during the experiments and separated into (1) the time needed to mount electrodes (‘Mounting time’, from being seated on the examination bench until all electrodes were mounted properly, including signal quality check on EEG electrodes); (2) the time needed to calibrate the system (‘Calibration time’, including instructions for calibration procedure, calibration data acquisition, and calibration of the BCI, the resistance of ROBERT^®^, and the amplitude of FES); (3) the ‘Total setup time’ (Mounting time + Calibration time); (4) the time spent exercising; (5) the ‘Total time’ used for exercising 60 min (‘Total setup time’ + exercise time); and (6) the time spent per repetition (‘Total time’/no. of exercise repetitions).

The participants’ perception of the system was quantified as subscale medians and IQRs of the filled-out questionnaires. A Pearson correlation coefficient was calculated for all subscales of the questionnaires (TLX: Mental, Physical, Temporal, Performance, Effort, and Frustration; IMI: Interest, Perceived Competence, Effort, and Tension; SQ: Comfort, No Pain, Tiredness, and Enjoyment) and the total TLX score to investigate any correlation between the participants perception of the system on their performance using the BCI. Additionally, a Pearson correlation coefficient was calculated for gender and age to investigate any correlation between the participants’ demographics and their performance using the BCI.

All results are reported as individual participant values, averages ± SD, or the median (IQR) of all participants. All calculations were done in MATLAB (version R2023b) or SPSS version 29.

## 3. Results

### 3.1. System Performance

All 10 participants were able to trigger the FES robotic system through the BCI.

The average TPR of the BCI was 62.6 ± 9.2%, while the individual participants reached a TPR in the range of 41.0% to 75.0%. The average accuracies and latencies for each participant are presented in Table 1.

The average BCI latency was −595 ± 129 ms (range: −439 ms to −786 ms), i.e., the BCI was able to identify the movement intention of the participant more than half a second prior to the peak negativity of the MRCP, which on average occurred 452 ± 292 ms after the “GO” cue.

### 3.2. System Feasibility

#### 3.2.1. Time Spent Using the System

Throughout the 60 min sessions, the participants on average completed 121 ± 14 exercise repetitions, in 5–7 bouts of exercise.

During the sessions, the factor multiplying the threshold ranged from 0.7 to 3.8 and had to be changed from the default 1.5 for 6 out of 10 participants. In 5 of the 6 cases, the factors had to be adjusted down, as the system was too unresponsive, and in two cases, it had to be adjusted up, as it was too responsive. For one participant, the factor had to be initially adjusted up and subsequently down.

To spend one hour (55.6 ± 4.4 min) exercising with the system, an average of 31.5 ± 3.3 min was required for mounting electrodes, training the BCI, and setting up the Robotic resistance and FES amplitude. The average time spent per repetition during the knee extension exercise was 44 ± 5 s. All results related to the time spent using the system are shown in Table 2.

#### 3.2.2. Questionnaires

**Figure 5:**
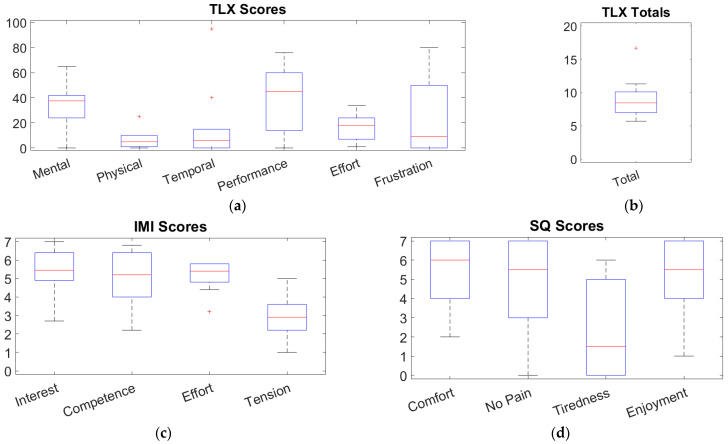
Boxplot summarizing the (**a**) TLX subscale scores; (**b**) TLX total score; (**c**) IMI scores; (**d**) SQ scores. For (**a**), the possible scores range from 0 to 100. For (**b**), the possible score ranges from 0 to 20. For (**c**,**d**), the possible scores range from 0 to 7.

The results of the questionnaires are summarized in Figure 5.

On average, the participants found that the system and exercising with it were mostly taxing in the performance and mental subscales of the TLX, with a median rating of 45.0 (46.0) and 37.5 (18.0), respectively (see Figure 5a). Additionally, there was a high level of variation in the participants’ perception of the frustration involved in the exercise, with a median of 9 (50). A single participant also found the temporal load of the exercise markedly taxing (adjusted rating of 95). The total TLX score indicates that the exercise constituted a moderately taxing exercise with a median rating of 8.5 (3.1) out of 20 possible (see Figure 5b).

In terms of the participants’ motivation for exercising using the system, the IMI found a moderate to high level of motivation (see Figure 5c). Overall, the IMI questionnaire shows a median motivation level of 5.0 (2.4), with the tension subscale (related to anxiety vs. comfort) being the least beneficial to motivation with a median score of 2.9 (1.4). The participants reported moderate to high levels of self-perceived competence and effort, with a median of 5.2 (2.4) and 5.4 (1.0), respectively, even though the achieved TPR did not exceed 70% for more than a single participant.

As for the SQ questionnaire, the results show moderate to high levels of positivity regarding the BCI-based exercise, despite a large variability in the participants’ responses (see Figure 5d). Participants reported a median comfort and enjoyment of 6.0 (3.0) and 5.5 (3.0), respectively, out of a total of 7. No pain and tiredness were rated at 5.5 (4.0) and 1.5 (5.0), respectively, indicating a low level of pain associated with the exercise and a small amount of tiredness being induced during the exercise.

The results of the investigation of the correlations between the BCI TPR and questionnaire subscales are shown in Table 3. There was no single subscale that was significantly correlated with the TPR of the BCI. However, it is worth noting that the subscale with the closest resemblance to a significant correlation (*p* = 0.122) was the TLX subscale ‘Effort’ with a negative correlation of −0.521.

Similarly, for the investigation of the correlations between BCI performance and demographics, the correlations observed were −0.192 (*p* = 0.595) and 0.343 (*p* = 0.331) for gender and age, respectively, and neither reached statistical significance.

## 4. Discussion

In this study, we presented the design of a hybrid rehabilitation system combining robotic-assisted resistive exercise with FES and BCI control and investigated the performance and feasibility of the system. The system allows a person on bedrest to actively initiate an exercise repetition using the BCI and engage their muscles using FES, even if they have little to no voluntary muscle control, which is essential for facilitating motor (re-)learning [9,10,11].

### 4.1. BCI Performance

The TPR of the utilized BCI was 62.6 ± 9.2%, which is relatively low in comparison with other studies utilizing similar EEG signals and applications (FES or robotics), where the TPR ranged from 67.15 to 86.30% [32,38,48,49,50]. The present study was conducted in a non-lab setting to simulate the environment that the system would eventually be used in (hospital or clinic), whereas the comparative studies seem to have been performed in laboratory settings [32,38,48,49,50], which are more favorable for obtaining quality EEG signals. Additionally, the present study utilized only five EEG electrodes (plus a reference and a ground electrode), limiting the EEG data available for processing, whereas the comparative studies utilized 9–64 (35.8 ± 27.3 on average) [32,38,48,49,50]. This was done to reduce the setup time, as the setup of intensive BCI systems (requiring more than 15 min to set up) is unacceptable [51]. It should be noted that EEG caps with 10 electrodes + references are possible to set up in less than five minutes, without EEG signal quality assurance [51]. The restriction to five EEG electrodes and the further reduction of the EEG into a single compound EEG signal may have negatively impacted the BCI performance. Including more EEG channels with individual signal processing and feature extraction should be investigated since it might yield a better BCI performance, albeit at a higher computational cost. Finally, comparative studies utilized more features [32,50] or relatively more advanced computational algorithms [48,49], which were avoided in the present study out of consideration for the practical usability of the system in a clinical setting, as these may require more elaborate BCI training, more education to be used, and more time to use in general. These above reasons can likely partially explain the worse BCI performance in the present study, but the considerations for accuracy versus complexity are necessary to consider for future studies and clinical feasibility.

### 4.2. Feasibility of the System

A general problem with BCI systems is the time spent preparing the system for use. The total setup time of the present system was assessed to be 33.0 ± 3.1 min. In a rehabilitation setting with limited therapist–patient time, this seems unfeasible. As reported by Jochumsen et al., 2020, the time spent setting up a BCI that should be used every day is ideally less than 15 min [51], which is a little less compared to the time spent in the present study to set up the BCI (mounting time: 10.6 min) and calibrate it (50 repetitions of 10 s = 8.3 min). Although Jochumsen et al., 2020 only referred to the use of a BCI and did not include a robot and an FES system needing calibration as well [51], it is unlikely that the therapists would approve a double setup time, even for a hybrid rehabilitation system. Similarly, McCrimmon et al., 2017 introduced a custom BCI headset, which took 10 min to set up and a further five minutes to calibrate, resulting in a total setup time of 15 min for the BCI alone [52]. It should be noted that this system did not include the attachment of a participant’s leg to a robotic device, nor the application of EMG and FES electrodes, the subsequent calibration of movement in the robot, or the FES stimulation parameters. Additionally, the system by McCrimmon et al., 2017 utilized only 25 BCI calibration samples (of 12 s, consisting of 6 s hand opening and 6 s hand closing) [52], whereas the present system used 50 (of 10 s), making the present system’s calibration data acquisition time 3.3 min longer. Thus, the present system’s mounting time and the BCI-specific calibration time are relatively comparable to other systems [52] and the desired setup time for BCIs [51], yet the complexity of the remaining hybrid system results in roughly twice the setup time. However, as the system targets patients who often will be unable to participate in other types of exercise, it may still be an attractive option. On the other hand, despite the system design being focused on simplicity, it might still be too complex and time-demanding to be used in the resource-limited rehabilitation field. Alternative solutions such as electrical stimulation systems capable of maintaining muscle mass while bedridden might be prioritized [53,54], despite these not being optimal for neural recovery, as they do not provide functional movement nor engage the user actively.

One way to reduce the setup time of the developed system would be to adopt dry electrodes rather than their wet counterparts. Multipin dry electrodes can be applied in as little as 5 min, although they may not be comfortable for extended use [55]. The mounting time of the BCI could thus be decreased by 5–6 min. Another important way to reduce the setup time of any BCI application is to introduce participant-independent BCI paradigms. This concept requires pre-calibrating the BCI using a data model previously obtained or designing it using demonstrably robust features across participants such as common spatial patterns [56] to bypass the need for calibrating BCIs prior to use and essentially creating a BCI that is ready for use immediately after mounting the electrodes [56]. This could decrease the calibration time of the present system by 11 min (out of the total 20.9 min used for calibration of the system). These changes would bring the total setup time down to 17 min, approaching the upper limit for setting up a BCI [51], and thus, it seems likely that therapists would accept such a setup time for a hybrid rehabilitation system as the one presented in the present study.

Overall, the system was found to be moderately taxing to use during exercise (total TLX: 8.5 (3.1)) and moderately-to-highly motivating and enjoyable to use (IMI: 5.0 (2.4), SQ enjoyment: 6.0 (3.0)). Likewise, the levels of pain and tiredness experienced by participants were favorable for the feasibility of the system. However, the responses to the SQ questionnaire, in particular, had considerable variability. This may be caused by the relatively few participants enrolled in this study or could simply be related to the subjective nature of the questions. Overall, the responses to questionnaires indicated that the system from a subjective point of view was feasible to use for exercise, although the results should be verified in a bigger study on a relevant target group.

It was interesting to see a tendency (not significant) for participants with high self-reports of ‘Effort’ (TLX) obtaining a relatively low BCI TPR. This may be an indication that those participants who found the system difficult to use (low TPR) would put more effort into using the system, which may be indicative of motivation and engagement. It is possible, however, that the reverse holds true, i.e., that those who put high effort into controlling the system unintentionally produced unfavorable EEG signals resulting in lower TPR performance. In relation to predictors for BCI performance, a previous large-scale study confirms our finding that gender does not influence the BCI performance [57], suggesting the validity of this result despite the low sample size.

### 4.3. Feasibility of Inducing Neuroplasticity

As with most neurorehabilitation, the overall goal of the present system was to rehabilitate neural damage through exploitation of neuroplasticity [58]. Although the BCI utilized in the present study had a comparatively low TPR, it obtained a TPR close to that of Niazi et al., 2012 (TPR: 67.15%), who demonstrated a neuroplastic effect following their intervention [38]. This suggests that the obtained BCI performance might be sufficient for inducing neuroplastic effects. However, as the aim for the present study was merely to demonstrate the technical performance and feasibility of the system, the ability of the system to drive neuroplastic changes and their assessment should be pursued in future work. It is proposed that such neuroplastic measures should be “complete” in assessing the neuroaxis, i.e., the descending pathways from the motor cortex to the lower limbs, the ascending pathways from the lower limbs to the sensory cortex, and the intrinsic circuits of the spinal cord, as suggested in the work by Leerskov et al., 2019 [59]. It should be noted that the latency of the BCI detections in the present study (595 ms prior to the anticipated peak negativity of MRCP) appears suboptimal, as the timing of the intervention relative to the MRCP has been found to be critical for neuroplasticity and optimal at 50 ms prior to the MRCP PN [60]. Previous studies have attempted to optimize the delay between the MRCP PN and the BCI detection, achieving latencies of −26 ms [61] and 315 ms [62] relative to the MRCP PN and onset of EMG, respectively. This suggests that there is room for improving the detection latency of the BCI used in the present study, but also, that this is not trivial considering the relatively large difference between latencies achieved in prior work [61,62]. Additionally, the latency of the BCI detections in the present work varied considerably between participants, which may indicate that the simplistic BCI design was insufficiently robust across participants in the setting of the experiment. This may further indicate the need for a future investigation of robust EEG processing techniques, features, and classifiers for the developed hybrid system in the relevant setting.

### 4.4. Limitations

This study was completed using ten healthy participants. It is unclear how the performance of the BCI would change if used on the target population; however, several previous studies have shown successful BCI applications for neurological patients [31,32,34,60,63,64], suggesting the feasibility of the technology for neurological patients. Likewise, the questionnaires used in this study seemed to indicate positivity towards exercising with the developed system. However, these results should be considered preliminary, as they were obtained only in able-bodied participants who may not accurately represent the opinions of a relevant patient group.

The BCI controls when FES will be administered. In an exercise setting, the accuracy of the BCI will determine the number of missed triggers and, inadvertently, how many exercise repetitions can be completed in a session. In this way, the BCI accuracy is a key factor in determining how effective the exercising is. Similarly, the BCI detection latency is found to be important for driving neuroplastic changes [60] and may thus impact the efficiency of the rehabilitation. FES itself may eventually lead to fatigue and thus affect the possible exercise duration [20,21]. However, the addition of the robotic device will help compensate for this fatigue and thus extend the possible exercise duration [23,24]. Thus, the BCI remains the main limiting factor of the system. The robotic device itself is not affected by the BCI performance in the system, although the coupling of the BCI-triggered FES with the robotic movement is the foundation for the active participation in the present system, which is crucial for the improvement in the rehabilitation scheme as a whole [9,10,11]. Hence, improvements to the BCI, particularly the detection accuracy and latency, may be a key factor for improving the rehabilitation efficacy and reliability in the present system.

The calibration data obtained for the BCI in the present study were obtained by having healthy participants do a minor initiation of a movement. This was performed as it had previously been found that imagined movements of healthy participants would produce much smaller MRCPs compared to those of paraplegic individuals [65]. Hence, the minor initiation of movement in the present study had the purpose of generating MRCPs mimicking those expected in the relevant target population.

The use of the 3-2-1-GO cue in the present study limits the time when the system can be activated. Therefore, the autonomy of the person training is compromised. However, it also prevents a lot of false positive detections. As asynchronous BCIs even with very low false positive rates will eventually lead to false triggers, especially if there are several seconds between user attempts of performing an action, cues may be necessary with fast recurrent BCI decisions (as in this study). It would be a subject of another study to determine how frequently cues could be presented to users—to balance the volume of exercise they would achieve with the mental fatigue they may experience from rapidly presented cues.

Potentially, participant-specific characteristics such as activity level could have affected the individual performance achieved using the BCI. However, the present work did not include measures of daily activity, and hence, no such analysis could be performed.

The manual adjustment of the classification threshold could easily lead to inconsistencies in a clinical setting. Replacing the adjustment with a more robust classification or automated procedure should be sought for consistent functioning.

Finally, despite active participation being enforced by the use of a BCI, no direct measurement was made to evaluate whether the participants had a sense of participation in the exercise. The IMI questionnaire encompasses the subscales “Effort” and “Perceived Competence,” which relate to the participants’ own sense of how much effort they put into the exercise and how well they performed, but a questionnaire asking specifically whether they felt as if they were actively participating or if they felt in charge of the exercise was not included. Additionally, participation could potentially be indirectly assessed physiologically as the level of attention/concentration derived from participants’ EEG data [34]. This, however, was not possible to assess with the present study’s EEG setup due to the larger number and different placement of EEG electrodes commonly used for attention detection [66,67,68].

### 4.5. Future Studies

In future studies, the system should be tested on a relevant group in need of neurorehabilitation, e.g., stroke or SCI populations. This test should be preceded by an assessment and implementation of any patient group-specific alterations in the system to ensure that the system is capable of safely accommodating exercise in the specific patient group investigated. It should be noted that robotics, FES, and BCIs have been previously used safely in stroke and SCI populations, both independently [1,25,39,47,50,64] and in combination [1,23,26,27,28,30,32,33,34,38,60]. The present system integrates all these technologies (robotics, FES, and BCI). It is speculated that the combination of the technologies poses no more risk to patients than systems combining only robotics and FES, as these are the “power producing” and moving parts of the system, whereas the BCI acts only as a trigger to facilitate active participation. Additionally, the system was equipped with safety measures in its current form. FES could only be triggered at specific times, which were made clear to the user through visual cues (see Section 2.2.3 and Section 2.2.4), and could be manually terminated by the experimenter if necessary (see Section 2.1.1). Furthermore, a safe mode was implemented in the robotic device to accommodate unwanted movement patterns/interaction forces (see Section 2.1.1). Yet, the present system should undergo assessments for its safe use in stroke or SCI patients, particularly due to the potentially limited sensory feedback and presence of spasticity [3,9], since the present study only evaluated the system in an able-bodied population.

Longitudinal studies with continuous neuroplastic assessments should be conducted in patients to provide evidence of the efficacy of the system as a neurorehabilitative tool and show if beneficial functional/neuroplastic changes can be attributed to the use of the system.

Improving the latency of the BCI triggering and the BCI TPR is desirable and should be pursued, but this should be achieved without adding complexity to the system. Furthermore, the overall setup and calibration time should be optimized, and if achieved, this will underline the feasibility of the system as a neurorehabilitative tool.

Finally, it would be relevant to implement a similar state machine as the one in Leerskov et al., 2024 [36], making the system more generalizable. As such, even in the case of changes in the exercising individual’s movement-producing capabilities (with or without FES), the user would have the opportunity to exercise using more of their own resources for improved rehabilitative outcomes [36].

## 5. Conclusions

The present study has shown the usability of the developed hybrid rehabilitation system in all attending participants, successfully combining robotic resistive exercise with FES and BCI control. The system is designed to be used for individuals with little to no muscular control to actively engage and initiate exercising, which is key for neural recovery [9,10,11]. In 10 able-bodied participants, the BCI detected movement initiation with a TPR of 62.6 ± 9.2 and a delay of −595 ± 129 ms. The system took 31.5 ± 3.3 min to prepare for use, which may be acceptable for patients with limited alternative options for active participation in exercise. The developed system was moderately taxing to use (TLX), moderately-to-highly motivating and enjoyable (IMI), while having favorable pain and tiredness scores (SQ) and was hence deemed feasible for use.

This study highlights that hybrid rehabilitation systems are complex to set up and calibrate, requiring considerable time, which is a scarce resource in the clinical settings where these systems are relevant. This should be considered when developing hybrid rehabilitation systems as this is a critical point for the adoption of the technology into practice. By extension, it is recommended that reporting the setup and calibration time becomes standard practice for hybrid rehabilitation systems, as these are inconsistently reported.

## Figures and Tables

**Table 1:** System performance results. BCI TPR; BCI latency—latency from BCI detection to MRCP peak negativity; a negative number means that the BCI trigger happened prior to the MRCP peak negativity.

Participant	BCI TPR (%)	BCI Latency (ms)
1	75.0	−737 ± 690
2	41.0	−751 ± 727
3	85.7	−554 ± 533
4	64.9	−511 ± 579
5	67.7	−489 ± 563
6	66.7	−547 ± 773
7	57.8	−439 ± 325
8	68.3	−470 ± 648
9	59.4	−786 ± 672
10	66.3	−670 ± 542
Average	62.6 ± 9.2	−595 ± 129

**Table 2:** Time spent using the system. Mounting time—the time for positioning electrodes and ensuring proper electrode impedance (≤10 kOhm); Calibration time—the time for recording MRCP trials, BCI training, finding the proper resistance of ROBERT^®^, and stimulation amplitude of FES; Total setup time—the total time spent on setting up the system (Mounting time + Calibration time); Exercise time—the time spent by the participant in the exercise trial; Total time—sum of time spent using the system. Per repetition—the time spent per exercise repetition given the total time spent using the system and the number of repetitions performed. All results are in seconds.

Participant	Mounting Time (min)	Calibration Time (min)	Total Setup Time (min)	Exercise Time (min)	Total Time (min)	Per Repetition (s)
1	13.1	17.3	30.4	44.2	74.6	97
2	9.5	18.1	27.6	60.4	88.0	53
3	13.6	24.7	38.3	52.7	91.0	46
4	11.2	24.8	36.0	58.8	94.8	47
5	9.5	22.2	31.7	55.5	87.2	40
6	12.3	20.0	32.3	59.3	91.6	39
7	9.3	21.9	31.2	54.4	85.6	51
8	9.0	20.4	29.4	56.8	86.2	37
9	10.0	21.7	31.7	58.5	90.2	45
10	8.5	18.3	26.8	55.3	82.1	41
Average	10.6 ± 1.7	20.9 ± 2.5	31.5 ± 3.3	55.6 ± 4.4	87.1 ± 5.4	44 ± 5

**Table 3:** Pearson correlation and respective significance levels for the correlations between participants’ BCI accuracy and their questionnaire subscale results.

Questionnaire	Subscale	Pearson Corr. (*p*-Value)
TLX	Mental	−0.351 (0.320)
	Physical	0.194 (0.591)
	Temporal	−0.022 (0.952)
	Performance	0.065 (0.858)
	Effort	−0.521 (0.122)
	Frustration	0.012 (0.975)
	Total	−0.217 (0.547)
IMI	Interest	0.330 (0.351)
	Perceived Competence	0.384 (0.274)
	Effort	−0.195 (0.589)
	Tension	0.023 (0.951)
SQ	Comfort	−0.111 (0.760)
	No Pain	−0.189 (0.602)
	Tiredness	−0.214 (0.552)
	Enjoyment	0.442 (0.201)

## Data Availability

The datasets presented in this article are not readily available because the consents provided by participants does not cover the use of the obtained data in research beyond the scope of the present study.
